# Modest effect of statins on fasting glucose in a longitudinal electronic health record based cohort

**DOI:** 10.1186/s12933-022-01566-w

**Published:** 2022-07-14

**Authors:** Tanushree Haldar, Akinyemi Oni-Orisan, Thomas J. Hoffmann, Catherine Schaefer, Carlos Iribarren, Ronald M. Krauss, Marisa W. Medina, Neil Risch

**Affiliations:** 1 Institute for Human Genetics, University of California San Francisco, 513 Parnassus Ave., San Francisco, CA USA; 2 Department of Clinical Pharmacy, University of California, San Francisco, CA USA; 3 Department of Epidemiology and Biostatistics, University of California, San Francisco, CA USA; 4 Kaiser Permanente Division of Research, Oakland, CA USA; 5 Departments of Pediatrics and Medicine, University of California, San Francisco, CA USA; 6 Department of Pediatrics, Division of Cardiology, UCSF, San Francisco, CA USA

**Keywords:** Statin, Fasting glucose, Cohort study

## Abstract

**Background:**

Prior studies of the glycemic effect of statins have been inconsistent. Also, most studies have only considered a short duration of statin use; the effect of long-term statin use on fasting glucose (FG) has not been well examined. The aim of this work is to investigate the effect of long-term statin exposure on FG levels.

**Methods:**

Using electronic health record (EHR) data from a large and diverse longitudinal cohort, we defined long-term statin exposure in two ways: the cumulative years of statin use (cumulative supply) and the years’ supply-weighted sum of doses (cumulative dose). Simvastatin, lovastatin, atorvastatin and pravastatin were included in the analysis. The relationship between statin exposure and FG was examined using linear regression with mixed effects modeling, comparing statin users before and after initiating statins and statin never-users.

**Results:**

We examined 593,130 FG measurements from 87,151 individuals over a median follow up of 20 years. Of these, 42,678 were never-users and 44,473 were statin users with a total of 730,031 statin prescriptions. FG was positively associated with cumulative supply of statin but not comulative dose when both measures were in the same model. While statistically significant, the annual increase in FG attributable to statin exposure was modest at only 0.14 mg/dl, with only slight and non-significant differences among statin types.

**Conclusions:**

Elevation in FG level is associated with statin exposure, but the effect is modest. The results suggest that the risk of a clinically significant increase in FG attributable to long-term statin use is small for most individuals.

**Supplementary Information:**

The online version contains supplementary material available at 10.1186/s12933-022-01566-w.

## Introduction

Statins (3-hydroxy-3-methylglutaryl-CoA reductase inhibitors) are prescribed widely to reduce low-density lipoprotein (LDL) cholesterol levels and risk of cardiovascular disease [1]. However statins may have adverse side effects in at least some individuals, including increasing the risk of new-onset diabetes mellitus (NOD). Previous studies based on both randomized controlled trials (RCT) [2, 3] and observational cohorts [4] have evaluated this risk. These studies vary widely in design, methods, type of statin prescribed and the length of follow up. A few of them have found little to no evidence of an association between statin use and the risk of NOD [2, 5]. For the most part, the studies with large sample sizes have reported statistically significant increases in the risk of NOD particularly in simvastatin and atorvastatin users [6, 7]. By contrast, very few studies have reported a significant increase in NOD with lovastatin use [8, 9]. The ascertainment of NOD also has varied widely across different studies, and the observed degree of association with statin use can depend on the method of ascertainment [10].

Although NOD diagnosis is typically based on FG levels, there are very few studies that have evaluated the effect of statin use on FG itself [8, 11]. One study [10] reported a significant increase in the risk of NOD, and elevated 2 h glucose (2hPG) and glucose area under the curve of an oral glucose tolerance test with statin use, but only a nominal increase in FG level. Despite intended lifetime use of statins, to date there has been only one cohort study of long-term effects of statin use (follow up period of 10 years) on FG [8]. However, like many other cohort studies, statin users were compared to individuals who were never prescribed a statin during the observation period [9, 11], a design that can lead to confounding (as we detail below).

For the studies that have reported an increased risk of NOD with statin use, the timing of the associated increased risk also appears inconsistent, with some studies suggesting no time dependency [6, 12], and others finding the greatest increase soon after the start of statin use [3, 4].

The aim of the present study was to investigate the effect of long term statin exposure (up to 20 years) on FG in a large, diverse cohort of individuals with many years of follow up based in electronic health records (EHRs), all within the same health care provider system (KPNC).

## Methods

### Participants

Participants in the study comprised the KPNC Resource for Genetic Epidemiology Research in Adult Health and Aging (GERA) cohort, consisting of approximately 103,000 individuals with detailed longitudinal information on statin use and FG levels through continuous follow-up in EHRs for an average of 20 years (study interval of 1996 through 2018). All participants were part of the RPGEH cohort of KPNC, a pre-paid, integrated health care delivery system with over 4 million enrolled patients. Since 1995, KPNC has had comprehensive EHRs that include detailed information on all aspects of medical care, including complete records of all dispensed prescriptions (i.e. only prescriptions ordered by clinicians that were dispensed to patients, and excluding those not dispensed), standardly coded diagnoses of all conditions, all laboratory test results, and, beginning in 2006, measurement of body mass index (BMI) at routine medical visits. As part of the RPGEH, GERA cohort members completed a survey and provided a saliva sample for DNA in 2007–2010. Participants consented to the broad use of their EHR, survey, and genetic data for studies of genetic and environmental influences on health. The study was approved by both the Kaiser Permanente Northern California Institutional Review Board and UCSF Human Research Protection Program.

### Data extracted from EHR

To capture the natural progression of FG over time in both statin users and never-users, all FG measurements and statin dispensing records were extracted from the EHRs, along with BMI and the dispensing records of drugs known to alter FG levels (including medications indicated for diabetes and others known to affect FG and plasma lipids) (see Additional file 1).

### Statistical analysis

**Fig. 1:**
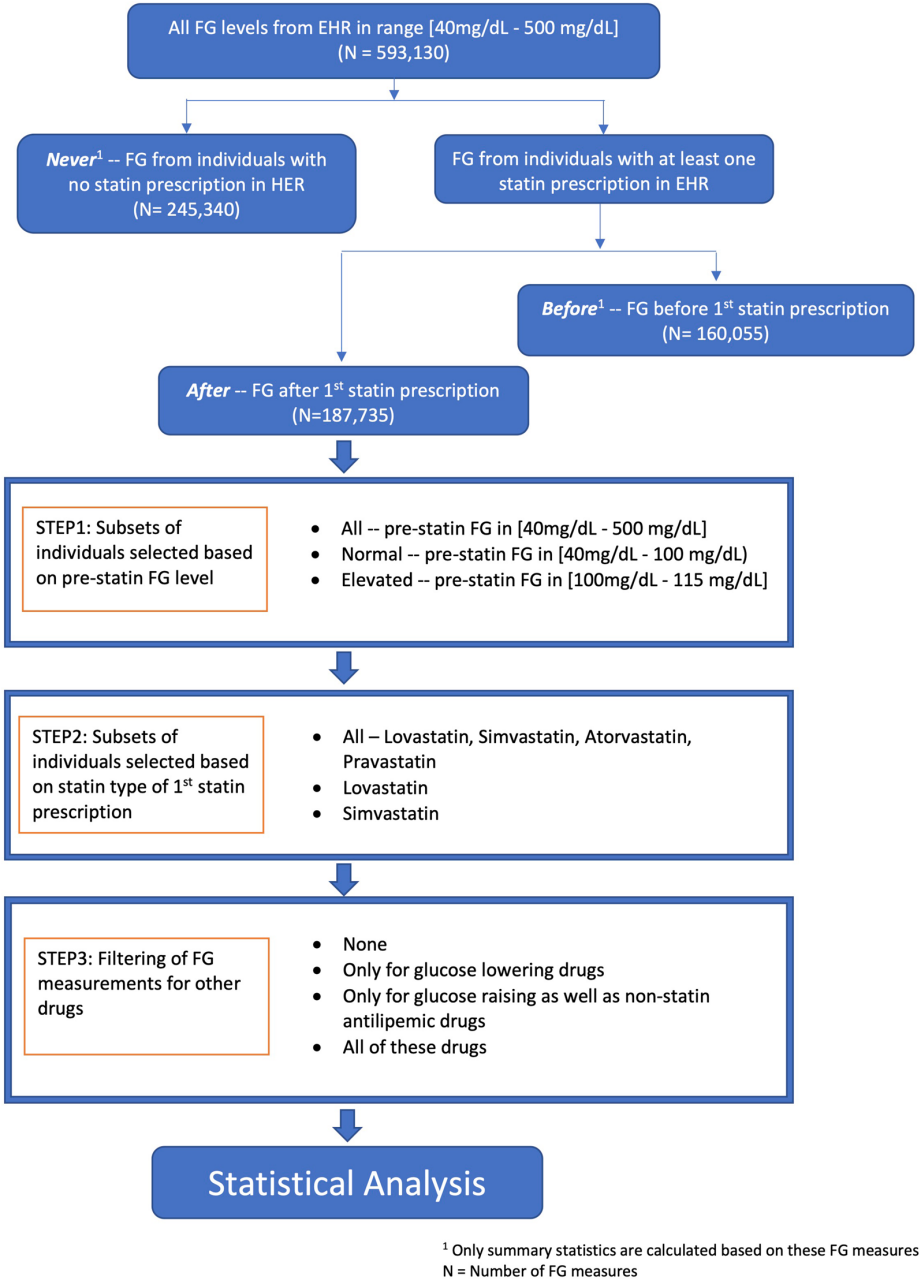
Flowchart illustrating the steps of selection of individuals and FG measurements for analysis

Our analysis focused on FG as the dependent variable, with covariates defined by characteristics of the individual in whom they were measured. To account for correlation of FG measurements within an individual, all analyses employed a linear mixed model approach (see Additional file 1). For analysis of the impact of statin use on FG, FG measurements were classified into three groups: those coming from individuals never prescribed a statin (“never statin”), those from individuals taking statins prior to their initiation of statins (“before statin”), and those from individuals taking statins during their statin usage (“during statin”) (Fig. 1).

As noted above, we considered effects of concurrent use of drugs that can lower or raise FG (Additional file 1: Table S1). Since removing (filtering) from analysis FG measurements when a patient is on these drugs may introduce bias (e.g. by excluding higher FG values), we conducted four similar analyses to address the potential impact of this bias: (1) no filtering; (2) filtering only for glucose lowering drugs (referred to as “diabetic drugs”); (3) filtering only for glucose raising as well as non-statin antilipemic drugs (referred to as “other drugs”); and (4) filtering for all of these drugs (Fig. 1). When filtering, we removed any FG measurement if the individual took a glucose altering drug (according to 2, 3 or 4 above) within 5 days of the FG measurement. With few exceptions, these therapies do not have a pharmacokinetic (e.g., half-life > 24 h) or pharmacodynamic (e.g., evidence of sustained glucose-raising effects following discontinuation) profile necessitating a washout period beyond 5 days in our study design [13]. The overall numbers of prescriptions of each drug and the numbers of FG measures filtered out for each are provided in Additional file 1: Table S1.

To further assess how the filtering of FG measurements may have affected the results, we repeated the analysis including only the subset of individuals who had a normal FG level (< 100 mg/dl) before the start of statin use, and who would thus be less likely to be prescribed a glucose lowering drug within the study period. An individual was considered to have a normal FG level before the start of statin treatment if all FG measures in the 2 years prior to the start of statin treatment were < 100 mg/dl. Similarly, we also conducted an analysis on the subset of individuals who had an elevated but non-diagnostic FG level (100–115 mg/dl) in the 2 years before the start of statin use (Fig. 1). For both of these groups, the likelihood of incident use of glucose-lowering medication is low, and thus would be subject to minimal FG measurement filtering.

## Results

### Final cohort and measurements for analysis

The final cohort included 87,151 individuals who had at least one eligible FG measurement. Of these, 51,481 (59%) were female and 35,670 male (41%). A total of 44,473 (51.0%) were statin users; a greater proportion of females were statin users (57%) than males (42%) (Table 1). Individuals self-reported their race and/or ethnicity as 80.8% white (WHT), 8.3% Latino/Hispanic (LAT), 7.3% East Asian (EAS), 3.1% African American (AFR) and 0.5% South Asian (SAS) [14]. The proportion of statin users varied modestly across the race/ethnicity groups, with most around 55%, but 51% for Whites and lowest for African Americans at 45%. Median age at the start of EHR for statin users (56 years) was greater than for the statin never-users (47 years), although the duration of EHR did not differ between the users and never users, with a median slightly greater than 20 years (Table 1).

**Table 1:** Demographics of study sample

Characteristic	Group	Statin user	Never-statin user	All
Sex	Female	29,356	22,125	51,481
	Male	15,117	20,553	35,670
	All	44,473	42,678	87,151
Race/ethnicity	African American	1221	1472	2693
	East Asian	3551	2853	6404
	Latino/Hispanic	3901	3324	7225
	South Asian	220	180	400
	White	35,580	34,849	70,429
Age	Start of EHR	55.96 (49.27–63.6)	47.27 (38.29–56.42)	52.05 (43.44–60.7)
	End of EHR	75.39 (68.67–82.66)	65.95 (56.88–75.33)	71.18 (62.35–79.91)
Length of EHR		20.7 (17.55–20.72)	20.38 (15.8–20.72)	20.63 (16.63–20.72)

Ages and length of EHR in years; medians and interquartile ranges

Among statin users, a total of 730,031 statin prescriptions were included in the analysis: lovastatin (65.2%), simvastatin (27.4%), atorvastatin (6.5%) and pravastatin (0.9%); the average number of statin prescriptions per individual was reasonably similar across sex and race/ethnicity, with males and Whites having slightly more (Additional file 1: Table S2). A total of 593,130 FG measurements among the 87,151 participants were available for analysis, an average of 6.8 FG measurements for each cohort member over the study interval (Table 2). This group and their measurements constituted the final cohort for analysis.

**Table 2:** Summary of FG measures in different groups

Trait	Group	Number of subjects	Number of FG measures	FG level (median mg/dl ± IQR)	Age at time of FG measure (median ± IQR)
Sex	All	87,151	593,130	97 ± 16	64.5 ± 15.9
	Female	51,481	325,084	95 ± 14	63.6 ± 16.3
	Male	35,670	268,046	99 ± 18	65.4 ± 15.3
Age	[21,40]	8294	16,811	90 ± 12	35.8 ± 6.2
	(40,50]	23,247	53,946	93 ± 13	46.4 ± 4.6
	(50,60]	44,328	139,694	96 ± 16	55.8 ± 4.8
	(60,70]	49,487	196,287	98 ± 16	65.1 ± 4.9
	(70,80]	32,416	140,695	99 ± 16	74.2 ± 4.8
	(80,90]	12,012	45,697	99 ± 17	83.1 ± 3.9
Race/ethnicity	African American	2693	18,511	97 ± 21	61.7 ± 15.7
	East Asian	6404	47,561	98 ± 17	60.9 ± 16.8
	Latino Hispanic	7225	47,719	98 ± 18	60.6 ± 17.5
	South Asian	400	3017	99 ± 21	57.3 ± 18
	White	70,429	476,322	97 ± 16	65.3 ± 15.4
Statin user	Never	44,473	245,340	94 ± 12	61.5 ± 17.6
	Before	39,552	160,055	98 ± 17	63.0 ± 13.9
	During	35,332	187,735	101 ± 22	68.9 ± 13.9

As expected, median FG was higher for males than females (99 versus 95 mg/dl, respectively; Table 2), although males were slightly older than females. FG increased continuously with age, with a median FG of 94.4 mg/dl for those aged 21 to 40 to 99.0 mg/dl for those aged 80 to 90. FG differed only modestly between race/ethnicity groups (Table 2). The association of FG (normalized) with age, sex, race/ethnicity, and BMI within all three groups of FG measurements (“Never Statin”, “Before Statin” and “During Statin”) was statistically significant according to the mixed effects statistical model (Additional file 1: Table S3). The FGs of men were significantly higher than those of women in all three groups (explaining about 3% of the variance), and also significantly increased with age (explaining 2–8% of the variance) and BMI (explaining 7 to 10% of the variance) (Additional file 1: Table S3). We also noted that FG levels of South Asians, East Asians and Latinos were higher than those of Whites and African Americans, especially among statin users. FG of African Americans was slightly lower or similar to that of the Whites. While the largest and most significant difference was observed for East Asians, it accounted for only 0.6% to 1.5% of the variance (R^2^), and the other race/ethnicity comparisons explained less (Additional file 1: Table S3).

### Comparison of “Never Statin”, “Before Statin” and “During Statin” FG measures

**Fig. 2:**
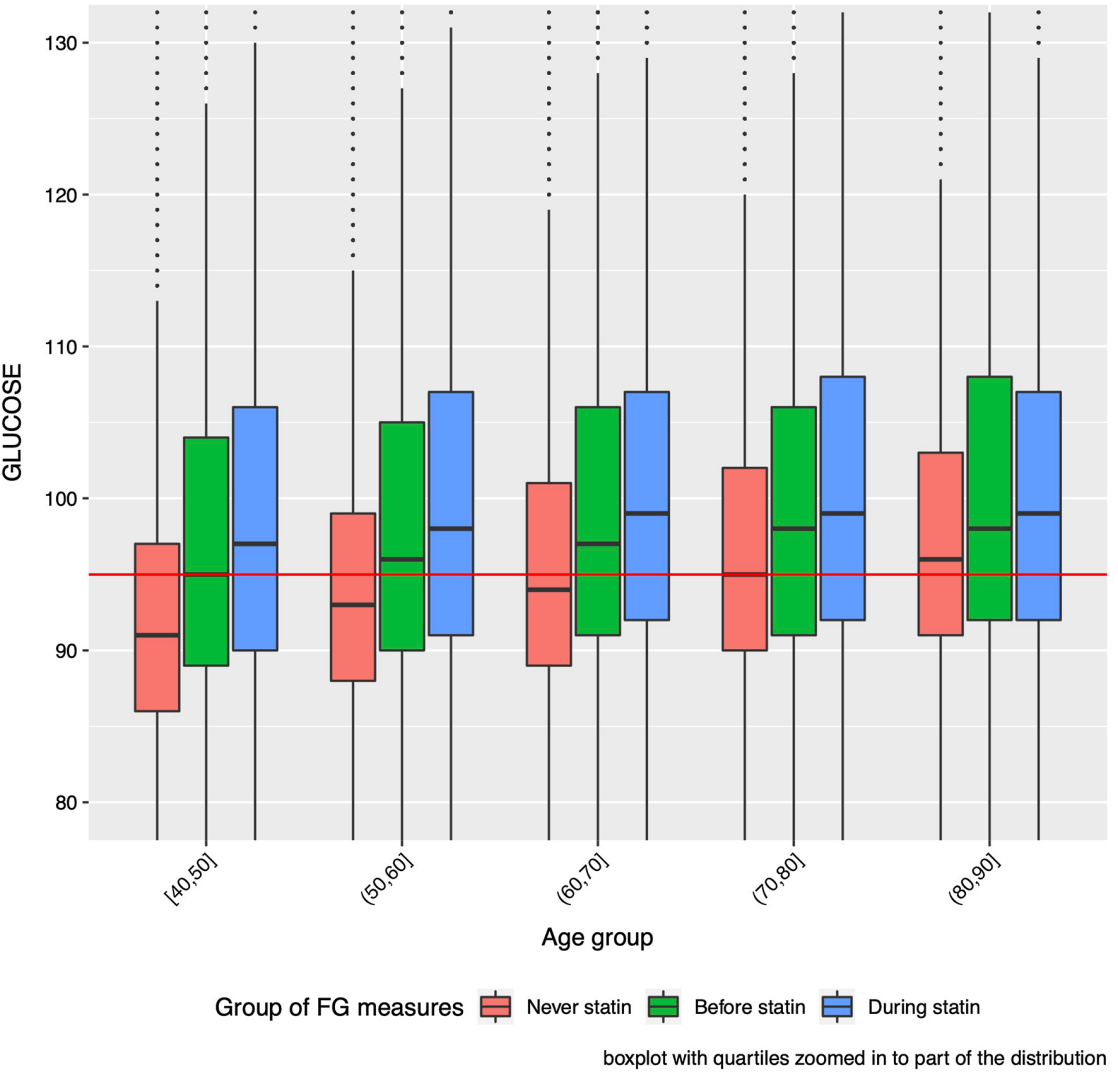
Distribution of FG (mg/dl) across different age groups for three subgroups of FG measures, filtered for all drugs

We then compared FG measurements between the “Before Statin” and “Never Statin” groups of FG measurements (Fig. 1). The median FG for the “Before Statin” group (98 ± 17 mg/dl, N = 160,055) was greater by 4 mg/dl than the median for the “Never Statin” group (94 ± 12 mg/dl, N = 245,340; p < 10^–203^) but less by 3 mg/dl than the median for the “During Statin” group (101 ± 22 mg/dl, N = 187,735; p < 10^–15^) (Table 2), suggesting that statin users have elevated FG prior to starting their statin treatment, but also have increased FG after initiating statins. However, since the median age at FG measurement for the “Before Statin” group (63.0 years) was also greater than that for the “Never Statin” group (61.5 years) and less than that for the “During Statin” group (68.9 years) and FG increases with age (Table 2), we further compared the distribution of FG among “Never Statin”, “Before Statin” and “During Statin” groups stratified into age at FG intervals, after filtering for all drugs (Fig. 2). We observed that within every 10-year age interval between 40 and 90, the median FG in the “Before Statin” group was greater than that in the “Never Statin” group, and less than that in the “During Statin” group (Fig. 2). The difference between the “Before Statin” and “Never Statin” groups attenuated with age, with a median difference of 4 mg/dl for those between 40 and 50, decreasing to a median difference of 2 mg/dl for those between 80 and 90, and about 3 mg/dl across all ages. The difference between the “During Statin” and “Before Statin” groups was less, and also attenuated with age, starting with a median difference of 2 mg/dl for those 40–50, decreasing to 1 mg/dl for those 80–90, and averaging about 1.5 mg/dl across all ages. We observed the same pattern when filtering for diabetes drugs only, filtering for other drugs (glucose raising and non-statin antilipidemics), or filtering for no drugs at all (Additional file 1: Figure S1). The differences observed when filtering for diabetes drugs were quite similar to those observed in Fig. 2 when filtering for all drugs; however, with no filtering or filtering for other drugs, the differences within age groups was larger between the “During Statin” and “Before Statin” groups, at about 3 mg/dl (Additional file 1: Figure S1). These differences also persisted after controlling for relevant covariates (BMI, sex, race/ethnicity), within each age interval and were statistically significant (Additional file 1: Figure S2).

### Association of FG with duration of statin exposure based on all statin users

While we found a modest overall increase in FG among statin users during versus prior to statin use, this did not account for duration of statin use. In this study, duration of statin use was characterized as cumulative supply (number of years of filled statin prescriptions; see Additional file 1 for definition). To evaluate different statins, for statin users, if they switched statins during the course of their treatment, we censored their data at the point of the switch. The distribution of total years of statin exposure for this analysis is given in Additional file 1: Figure S3; 32.1% (N = 11,343 subjects) had a total duration of use of at least 5 years; 8.1% (N = 2865 subjects) had a total duration of at least 10 years; 0.5% (N = 192) subjects had a total duration exceeding 15 years. To assess the longitudinal impact of statin use, we first used a mixed effects model to adjust FG for covariates; FG measures were normalized and then adjusted for age, BMI, sex, and race/ethnicity as well as the average FG level in the 5 years prior to statin initiation (Additional file 1: Table S4), since the FG level of an individual after the start of statin will be related to the FG level of that individual before the start of statin. Next, we used linear regression to study the association of FG level (normalized and adjusted for covariates) with cumulative supply of statin up to the point of that FG measurement (see “Methods”). FG levels were positively associated with cumulative supply, and the association remained significant (p-value < 10^–16^) after filtering for each of the three other drug treatment categories (diabetic, other, or both) (Table 3). We repeated the same analysis for cumulative dose (see “Methods”). Cumulative dose was also positively associated with FG levels and the association remained significant (p-value < 10^–10^) irrespective of filtering for other drugs. When both cumulative supply and cumulative dose were included in the same regression model, the p-value for cumulative supply remained highly significant, while the p-value for cumulative dose was greatly attenuated and either non-significant or only marginally so (Table 3). We also note that filtering for other FG-influencing drugs did not impact the regression coefficients for cumulative supply, while filtering for diabetic drugs enhanced the regression coefficient for cumulative supply and statistical significance. This suggests that the strong effect of diabetic drugs on FG diminished the relationship of FG with statin supply, as might be expected.

**Table 3:** Association of statin exposure (cumulative supply, cumulative dose) with normalized FG based on FG measurements from all statin users with various FG-influencing drug filters

Filter	Univariate model				Multivariate model			
	Cumulative supply		Cumulative dose		Cumulative supply		Cumulative dose	
	Regress coeff (s.e.)	p-value (R^2^)	Regress coeff (s.e.)	p-value (R^2^)	Regress coeff (s.e.)	p-value (R^2^)	Regress coeff (s.e.)	p-value (R^2^)
None n = 157,381	0.00373 (0.00041)	2.47E−19 (0.0005)	0.00214 (0.00031)	9.44E−12 (0.0003)	0.00323 (0.00053)	1.49E−09 (0.0005)	0.00060 (0.00041)	1.41E−01 (0.0005)
Diabetic n = 137,629	0.00523 (0.00041)	4.20E−37 (0.0012)	0.00296 (0.00032)	1.29E−20 (0.0006)	0.00468 (0.00053)	1.06E−18 (0.0012)	0.00067 (0.00041)	1.04E−01 (0.0012)
Other n = 121,015	0.00392 (0.00046)	1.42E−17 (0.0006)	0.00243 (0.00036)	1.24E−11 (0.0004)	0.00326 (0.00060)	4.55E−08 (0.0006)	0.00081 (0.00047)	8.39E−02 (0.0006)
All n = 106,913	0.00512 (0.00046)	5.38E−29 (0.0012)	0.00324 (0.00037)	9.66E−19 (0.00070)	0.00428 (0.00060)	6.64E−13 (0.0012)	0.00105 (0.00048)	2.70E−02 (0.0012)

R^2^ in multivariate model is for both cumulative supply and cumulative dose combined

### Association of FG with statin exposure in two subgroups of statin users

Next, we repeated the analysis for two subgroups based on normal (FG < 100 mg/dl) or elevated (100 < FG < 115 mg/dl) FG prior to the initiation of statin use. Comparing the regression coefficients of FG on cumulative supply in the normal FG pre-statin subgroup to the entire group (Table 4), the association of FG with cumulative supply was more significant and the regression coefficient was larger (approximately double, 0.00976 vs 0.00512) for the normal group.

**Table 4:** Association of statin exposure (cumulative supply) with normalized FG from all statin users and those with normal FG (< 100) within 2 years prior to statin initiation, by statin type^b^, filtered for all drugs

Prior FG	Statin Type	Number of individuals	Number of observations	Regress coeff (S.E.)	p-value (R^2^)
All	All	25,134	106,913	0.00512 (0.00046)	5.38E−29 (0.0012)
	Simva	8166	31,681	0.00546 (0.00108)	4.04E−07 (0.0008)
	Lova	14,327	69,222	0.00463 (0.00053)	4.88E−18 (0.0011)
	Atorva	2339	5231	0.00656 (0.00327)	4.52E−02 (0.0008)
Normal (< 100)	All	11,496	49,415	0.00976 (0.00069)	2.19E−45 (0.0040)
	Simva	3765	14,471	0.00908 (0.00168)	7.21E−08 (0.0020)
	Lova	6542	32,309	0.00883 (0.00080)	2.43E−28 (0.0038)
	Atorva	1051	2280	0.01245 (0.00485)	1.02E−02 (0.0029)

^a^Adjusted for covariates age, sex, BMI, race/ethnicity and average FG level during the 5 years prior to start of statin

^b^Adjusted for covariates age, sex, BMI, race/ethnicity and average FG during the 5 years prior to start of statin

**Fig. 3:**
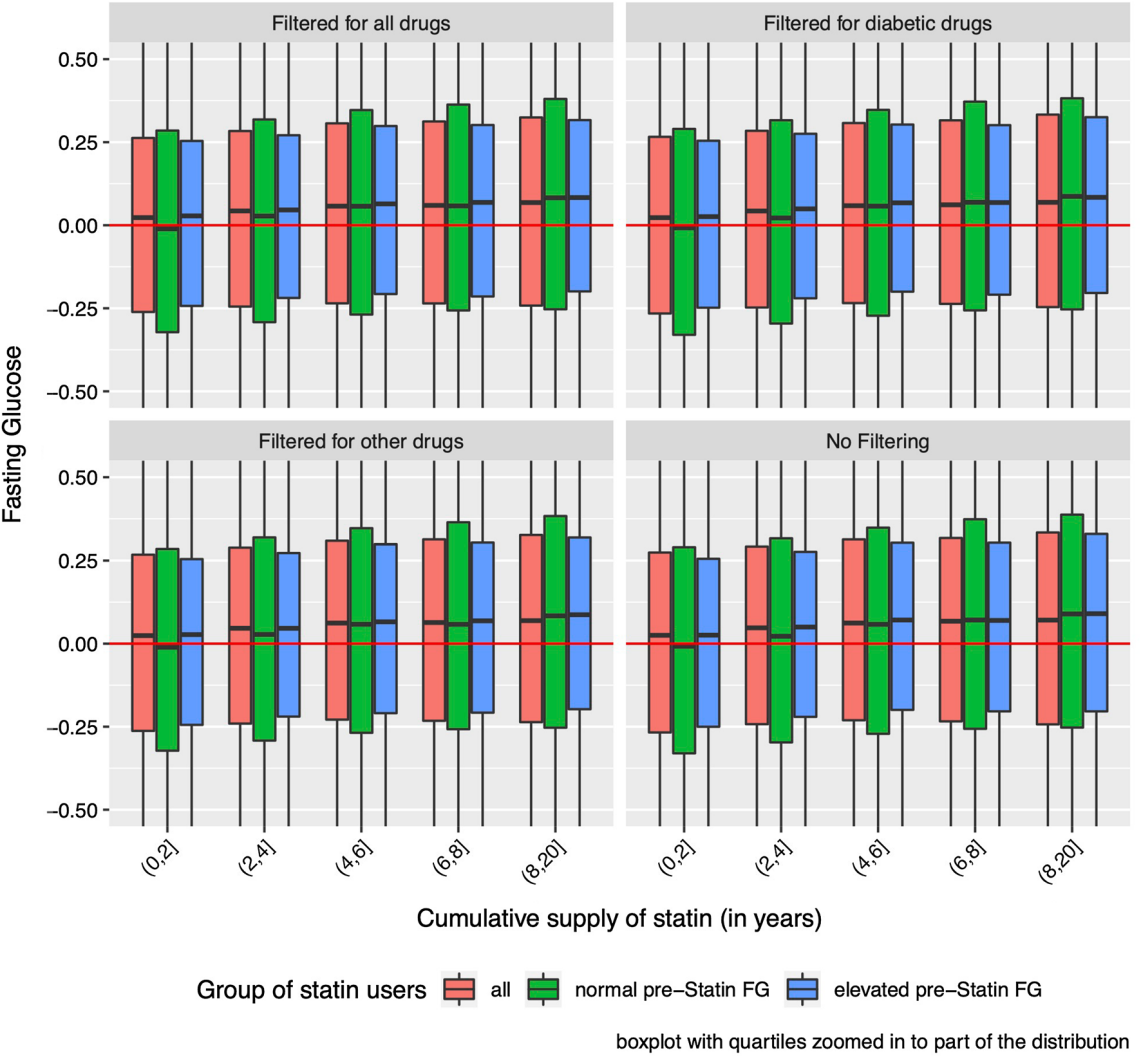
FG by duration of statin exposure for all statin users and two sub-groups; adjusted for age, sex, BMI, race/ethnicity and average FG during the 5 years prior to start of statin

We then examined the pattern of increase of FG adjusted for covariates with cumulative supply. Here the increase of FG with years of statin use was linear throughout the range of years, and that pattern was the same for all FG filtering models and between the normal and elevated FG groups (Fig. 3).

### Association of FG measures with statin exposure measures for different statin types

We repeated all the analyses for three subgroups of FG measurements based on statin type: lovastatin, simvastatin and atorvastatin. Considering all statin users and filtering for all drugs, all three statin types showed a significant regression on cumulative supply (Table 4). The regression coefficient for atorvastatin was slightly higher and for lovastatin slightly lower, but the differences were not statistically significant (Table 4). As was seen for all statins combined, when restricted to statin users with a normal FG prior to statin initiation, all regressions were significant, and the coefficients nearly double compared to all statin users (Table 4); the differences of regression coefficients across the statin types were small and not statistically significant (Table 5).

**Table 5:** Association of statin exposure (cumulative supply) with FG (no normalization)^a^, based on statin users who had normal FG prior to statin use, filtered for all drugs, by statin type

Statin type	Number of individuals	Number of observations	Regression coefficient (S.E.)	R^2^
All	11,496	49,415	0.135 (0.00954)	0.00403
Simva	3765	14,471	0.126 (0.02234)	0.00221
Lova	6542	32,309	0.122 (0.01111)	0.00371
Atorva	1051	2280	0.181 (0.08167)	0.00215

^a^Adjusted for covariates age, sex, BMI, race/ethnicity and average FG during the 5 years prior to start of statin

The pattern of change in FG with statin exposure across the statin types revealed a similar linear pattern with years of statin use across the three statin types and also as seen for all statins, after adjustment for covariates (Additional file 1: Figure S4).

## Discussion

### Long term effect of statin use on FG

In this study, we have extensively investigated the effect of long term statin use on FG in the large, ethnically diverse KPNC GERA cohort. We observed the usual strong effects of sex, age and BMI on FG. We also noted racial/ethnic differences, particularly in East Asians, who had higher FG, although explaining only at most 1.5% of the variance.

### Cumulative supply vs cumulative dose

Of note, in our regression analyses, the regression coefficients were stronger and more significant for cumulative supply than for cumulative dose, especially when both were included in a multivariate model. Since dose was defined based on LDL lowering (an effect primarily due to statin-induced suppression of hepatic cholesterol synthesis), the weaker relationship of the glycemic effect with dose suggests that the glycemic effect is due to either an extrahepatic effect (e.g., in beta cell or muscle) or to a mechanism unrelated to cholesterol synthesis. While an actual mechanism for the modest glycemic effect of long term statin use has not been established, a recent study has indicated that a statin-induced increase in insulin resistance may be responsible [15].

### Clinical impact

We observed that FG is significantly associated with statin exposure, overall and over time, with an apparent linear trend with years of use when adjusted for covariates. However, this trend was modest. Clinical impact of statins on FG can be determined by the predicted amount of change in FG with duration of statin exposure. It is challenging to universally estimate the size of effect of statin exposure on FG level based on observational cohort data because of treatment effects, in particular once the FG exceeds the level leading to a diagnosis of diabetes and its treatment. Filtering out FG measurements associated with the use of diabetes drugs may introduce bias in the analysis due to the exclusion of high FG measurements. On the other hand, if we do not filter for glucose lowering drugs, the FG measurements associated with those drugs are biased downwards. Hence, to gain a more accurate estimate, we only considered the subgroup of FG measurements from individuals who had a normal FG level before the start of statin use (as defined in “Methods”), as these individuals rarely were influenced by subsequent diabetic medications. Since here we are estimating the effect size, we did not normalize the FG level, but did adjust the FG measurements for the same covariates as before. With adjusted FG as the dependent variable in a regression model on cumulative supply, FG level increased on average 0.14 mg/dl per year (Table 5). Over a 10 year period, the expected increase would be 1.4 mg/dl, comparable to what we observed overall for statin users after versus before statin initiation. As suggested in the previous analyses, there were non-significant differences among the three statin types: 0.18, 0.13 and 0.12 mg/dl for atorvastatin, simvastatin and lovastatin, respectively. Our results suggest that, on average, given the small glycemic effect of statins on FG, the clinical impact of statin exposure will be low in individuals who do not have pre-statin FG levels near the threshold used to diagnose diabetes.

To the best of our knowledge, only one prior study has explored the change in FG due to long term statin exposure, which found a moderate increase of FG with statin use [8]. That study differed from ours in several important ways. First, it focused exclusively on an East Asian (Korean) population, while ours was ethnically diverse. However, more significantly, that study included FG measures of statin never-users in their comparison with statin users, which leads to confounding between statin initiation and statin use, and a potential bias.

The average FG among subjects who had no statin prescription in the EHR was significantly (p-value < 2.2 × 10^–308^) lower (by about 3 mg/dl, adjusting for age) than that among statin users prior to their first statin prescription (even after adjusting for relevant covariates). Perhaps this is not surprising as some indications for a statin prescription (e.g. elevated LDL-cholesterol, elevated BMI) are likely also associated with elevated FG. This observation suggests that FG among statin users should not be compared to that of subjects who never went on a statin, as elevated FG appears to be associated with the likelihood of receiving statin therapy as opposed to the actual exposure to statins. Thus, if this is not taken into account, it could appear that statin users are at increased risk of diabetes due to statin use, rather than being at increased risk prior to any statin use.

### Study limitations

Our study is an observational cohort study, distinct from an RCT. The advantage of an RCT is the comparability (due to randomization) of treatment and placebo arms to assess the direct effect of the drug, free of potential confounders that might influence an observational study of users and non-users. Indeed, in our study, we showed that statin initiation is a confounder of statin effects on FG, because individuals who took statins had higher FG prior to statin initiation compared to those who were never treated with statins. We avoided this bias by comparing FG measurements of statin users prior to versus during statin use. Our study provided additional advantages over typical RCTs in that it is population based, large and diverse in terms of patient characteristics; it allowed us to evaluate and adjust for significant covariates, and all subjects were members of a single health care system with standardized measurements of study variables such as FG and statin utilization; furthermore, study subjects were followed for much longer than is feasible in a typical RCT.

One limitation of our dataset is that there are a large number of individuals who were first prescribed lovastatin, and we have data for these individuals over a longer time period than for the other statins. In contrast, fewer individuals were prescribed atorvastatin as their first statin and the follow-up period was also shorter, as expected, since lovastatin came to market in 1987 while atorvastatin was not available until 1997. This reduced the relative number of atorvastatin users compared to lovastatin and simvastatin. Nonetheless, the numbers were sufficient to demonstrate a significant but modest glycemic effect of atorvastatin, and no difference among the statins. Thus, while there may be differences between lovastatin, simvastatin and atorvastatin in their glycemic effect (i.e., larger for atorvastatin and smaller for lovastatin), those differences are small, and the glycemic effect of atorvastatin is of little clinical significance, as it is for lovastatin. Thus, our conclusion of modest clinical impact applies to statins as a class.

Our results may also help explain inconsistencies in the literature regarding the effect of statins on FG and diabetes risk. With a modest overall effect, depending on the FG levels of the group examined, some studies would show increased risk for NOD, while others would not. It is also conceivable that the glycemic impact of statins is not uniform across individuals, even with the same starting FG levels, for reasons that may include genetic, metabolic, behavioral or environmental factors. Thus, some individuals may be more prone towards increased FG and diabetes risk than others; however, they are likely small in number and/or not at substantially increased risk, unless others are at reduced risk of NOD from statins, thus contributing to the modest overall increase in FG with statins.

## Conclusion

FG is associated with statin exposure, but the effect size is small. The results suggest that the risk for new onset diabetes attributable to statin exposure is small for individuals whose pre-statin FG level is not near the threshold for diagnosing this condition.

## Supplementary Information

**Additional file 1. **Supplementary Methods, Tables S1-S4 and Figures S1-S4.

## Abbreviations

FG: Fasting glucose
EHR: Electronic health record
LDL: Low-density lipoprotein
NOD: New-onset diabetes mellitus
GERA: Resource for Genetic Epidemiology Research on Adult Health and Aging cohort
RPGEH: Research Program on Genes, Environment and Health
KPNC: Kaiser Permanente Northern California
DDD: Defined Daily Dose
LMM: Linear mixed model
BMI: Body Mass Index
RCT: Randomized controlled trial
