# Supplementary material for: Modest effect of statins on fasting glucose in a longitudinal electronic health record based cohort
Source: Cardiovasc Diabetol. 2022 Jul 14;21:132. doi: 10.1186/s12933-022-01566-w (PMC9284686; doi:10.1186/s12933-022-01566-w)
Supplement: Supplementary file 1 — Additional file 1. Supplementary Methods, Tables S1-S4 and Figures S1-S4. [file 12933_2022_1566_MOESM1_ESM.docx]

**Additional File 1: Supplementary Methods, Tables S1-S4 and Figures S1-S4**

**Supplementary Methods**

Data extracted from EHR

Outpatient Fasting Glucose: All outpatient measurements of FG were performed by a central laboratory and the results automatically entered into the laboratory information system of the EHR. FG measurements obtained from individuals prior to their 21^st^ birthday and measurements occurring after their 90^th^ birthday were excluded. FGs less than 40 mg/dL or greater than 500 mg/dL were also excluded as outlier measurements. Because FG is highly skewed, all analyses report either medians and interquartile range (to keep the original scale) or normalized FG for statistical significance tests as described below.

Statin Prescriptions: Participants treated with statins had numerous statin prescriptions obtained from dispensing records from 1996 to 2018. Statin dose for each dispensed prescription was calculated using two independent sets variables :1) the number of days supplied and the total number of tablets dispensed, and 2) dose per day and number of tablets per dose. The daily dose in tablets per day was calculated using the two sources, and those that gave consistent results, with or without imputation (see Online Appendix), were retained for analysis [1]. Briefly, if two values for an individual were inconsistent, we (1) kept whichever was most common in the dataset (if present in 10% of initially consistent prescriptions), and then (2) kept one value if it was in the initially consistent prescriptions and the other value was not. If an individual had an inconsistent statin dose even after imputation, the participant was censored and all data from that time point onward were excluded from analysis.

The major statin types used in the cohort were lovastatin, simvastatin, atorvastatin and pravastatin; others were not included in the analysis due to insufficient numbers (other statin types and combinations including statins occurred in <0.2% of the population). Because statin types differ in lipid-lowering potency (i.e., average reduction in level of LDL cholesterol per mg dose of statin), a relative dose was determined based on the milligrams per day of drug prescribed and the statin type (i.e., simvastatin is approximately 0.5 times less potent than atorvastatin at the same milligram per day dose). Defined Daily Dose (DDD) was calculated as the relative dose times the number of tablets per day [2].

Two cumulative measures of statin medication exposure were calculated at a given time point (typically corresponding to the occurrence of a FG measure): (1) a cumulative supply measure determined by the number of days of all statin prescriptions filled up to that date; and (2) a cumulative dose, defined as the weighted sum of all the DDDs up to that time point, with the weight being the number of days of supply at that DDD. For both cumulative measures, number of days was divided by 365 to calculate yearly exposure. We only included individuals who initiated statin treatment during the study period. Thus, if the first fill of a statin prescription for an individual was within 6 months of the start of membership or the start of the EHR (1996), or if there was a gap > 6 months in membership just before the first statin prescription, the exact age of statin initiation was assumed to be unknown and thus these individuals were excluded. If there was a gap in KPNC membership of more than 1 year between two statin prescriptions, all data after the initial date of that gap were excluded from subsequent analysis of that individual. If the gap in membership was less than 1 year, the individual was assumed to have continued with the same statin type and dose during the gap. For individuals prescribed multiple types of statin over the observation period, only FG values prior to the first switch in statin type were included.

To evaluate the effect of statin on FG for each statin type, we categorized individuals by the statin type of their 1^st^ prescription (Figure 1). Since pravastatin was prescribed for a relatively small number of individuals, we excluded those individuals from the statin-specific analysis but they were included in the analysis of all statins.

Medications Affecting FG: Aside from the possible role of statins, other drugs lower (e.g., metformin, insulin) or raise (e.g., niacin, atypical antipsychotics, immunosuppressants) FG (Supplementary Table 1). Complete prescription data on FG lowering and raising medications were abstracted from the EHR and used to calculate the prescription periods for these medications in relation to measurements of FG in order to examine and control for possible effects of these medications on FG measurements. As described below, the analyses filtered FG measurements for the occurrence of different classes of these drugs.

Covariates: Since FG is highly correlated with BMI, BMI at the time point nearest to the date of the FG measurement was included as a covariate. If no BMI was available within 5 years of the date of that FG measurement, BMI was considered missing for that FG measurement. Gender, self-reported race/ethnicity, and age at the time of FG measurement were included as covariates in all except age-specific analyses.

Definition of Statin User: Statin users were defined as those with at least one statin prescription in the EHR, known age at statin initiation, and first statin prescription for any of the four included statin types. Statin never-users were those with no statin prescriptions in their EHR.

Classification of FG Measurements: FG measurements were categorized in terms of their occurrence in relation to statin prescriptions as “Never Statin” if obtained from individuals who were never prescribed statins during the study interval, “Before Statin” if the FG measure occurred among statin users prior to the initiation of statin treatment, and “During Statin” if the FG measure occurred within a window of statin treatment.

Statistical Analysis

Analyses were conducted using the linear mixed model (LMM) (lmerTest[3]) in R version 3.5.1 [4]. To satisfy the normality of the response variable in LMM, we normalized FG by an ordered quantile normalizing transformation (using the “bestNormalize” package [5] in R). All the graphs were prepared using ggplot2 [6]. Since analyses included multiple FG measures from a single individual over time, the LMM included a random effect term (as intercept) to allow for intra-individual correlation. Statistical significance was generally calculated by t-tests of regression coefficients from the LMM, with p<0.05 used for significance. We also calculated R^2^ values to assess the variance explained by covariates in the regression models.

Consistency Check of Statin Prescriptions

1. Number of tablets per day was calculated for each prescription using two sets of information
   1. total number of tablets dispensed (RXAMT) was divided by the number of days’ supply (RXSUP)
   2. number of tablets per dose (RXDOSE) was multiplied by dose per day (RXFREQ)
2. The group of prescriptions for which the number of tablets per day calculated by both methods were equal or approximately equal, were defined as “good” prescriptions. These prescriptions were considered as consistent without any imputation. There were a total 15,23,498 such prescriptions (86% of all 17,70,822 prescriptions).
3. For the rest of the prescriptions we imputed the value of one of the key variables (RXAMT, RXSUP, RXDOSE or RXFREQ) and considered the imputed prescription as consistent if the number of tablets per day calculated by both methods was equal or approximately equal (calculated after imputation). For imputation, we used the common values of the key variables among the “good” prescriptions (present in al least 10% of all “good” prescriptions), starting from the most common value to the least common value; 1,65,075 prescriptions were found consistent after imputation (9.3% of all prescriptions).
4. We also considered a prescription as consistent if the number of tablets per day calculated from method (a) existed among the “good” prescriptions (and number of tablets per day calculated from other method did not exist among the “good” prescriptions). Here method (a) was prioritized since the number of days’ supply was used to calculate statin exposure; 76,237 prescriptions were found consistent in this way (4.3% of all prescriptions).
5. 6012 prescriptions (0.34% of all prescriptions) remained inconsistent and were excluded.

[1] A. Oni-Orisan, T. Hoffmann, M. Medina, E. Jorgenson, C. Schaefer, R. Krauss, C. Iribarren, N. Risch, Validation of Electronic Health Records for the Assessment of Statin Dosing In Research, Journal of Clinical Lipidology. 11 (2017) 836–837. https://doi.org/10.1016/j.jacl.2017.04.104.

[2] A. Oni-Orisan, T.J. Hoffmann, D. Ranatunga, M.W. Medina, E. Jorgenson, C. Schaefer, R.M. Krauss, C. Iribarren, N. Risch, Characterization of Statin Low-Density Lipoprotein Cholesterol Dose-Response Using Electronic Health Records in a Large Population-Based Cohort, Circulation: Genomic and Precision Medicine. 11 (2018). https://doi.org/10.1161/CIRCGEN.117.002043.

[3] A. Kuznetsova, P.B. Brockhoff, R.H.B. Christensen, **lmerTest** Package: Tests in Linear Mixed Effects Models, Journal of Statistical Software. 82 (2017). https://doi.org/10.18637/jss.v082.i13.

[4] R Core Team, R: A Language and Environment for Statistical Computing, (2020).

[5] R.A. Peterson, J.E. Cavanaugh, Ordered quantile normalization: a semiparametric transformation built for the cross-validation era, Journal of Applied Statistics. 47 (2020) 2312–2327. https://doi.org/10.1080/02664763.2019.1630372.

[6] H. Wickham, ggplot2: Elegant Graphics for Data Analysis, Springer-Verlag New York, 2016.

**Supplementary Tables**

Table S1: Number of prescriptions of different drugs

| Drug Class | Drug Type | Drug Subtype | Number of Prescriptions | Number of FG^[[1]](#footnote-1)^ filtered out |
| --- | --- | --- | --- | --- |
| Diabetic drugs | Glucose lowering drugs | Insulin | 263596 | 18526 |
|  |  | Metformin | 308505 | 60380 |
|  |  | Sulfonylureas | 275352 | 50945 |
|  |  | Other Diabetes drugs | 54986 | 11416 |
| Other drugs | Glucose raising drugs | Adenocorticosteroids | 206970 | 8382 |
|  |  | Antipsychotics | 62893 | 5391 |
|  |  | Cyclosporine | 25108 | 1291 |
|  |  | Diuretics | 669265 | 114726 |
|  |  | HIV | 38196 | 2864 |
|  |  | Tacrolimus | 13067 | 1082 |
|  | Non-statin Antilipemic drugs | Ezetimibe | 47383 | 12162 |
|  |  | Fibrate | 85109 | 18145 |
|  |  | Other^[[2]](#footnote-2)^ | 74124 | 13294 |

Table S2: Summary of statin prescriptions in different groups

| Characteristic | Group | Number of Statin Prescriptions | Statin Prescriptions per subject  (median ± IQR) | Age at 1^st^ statin prescription  (median ± IQR) |
| --- | --- | --- | --- | --- |
| Sex | Female | 362910 | 12.0±21 | 65.1±14.7 |
|  | Male | 367121 | 13.0±22 | 65.3±14.4 |
| Race/ethnicity | African American | 22192 | 10.0±19 | 62.2±13.6 |
|  | East Asian | 47737 | 12.0±22 | 61.6±14.6 |
|  | Latino/Hispanic | 50197 | 10.0±19 | 62.1±14.6 |
|  | South Asian | 2711 | 8.5±20 | 58.9±15.0 |
|  | White | 607194 | 13.0±23 | 65.9±14.3 |
| Statin type | Atorvastatin | 47742 | 6.0±10 | 69.9±12.6 |
|  | Lovastatin | 476078 | 16.0±26 | 63.6±14.2 |
|  | Pravastatin | 6348 | 6.0± 9 | 66.6±15.7 |
|  | Simvastatin | 199863 | 12.0±21 | 65.5±14.4 |

Table S3: Association of covariates with normalized FG level, by statin use group of FG measurements, filtered for all FG-influencing drugs

|  | Statin User Group | | | | | |
| --- | --- | --- | --- | --- | --- | --- |
|  | Never | | Before | | During | |
| Covariate | Regress Coeff  (s.e.) | p-value  (R^2^) | Regress Coeff  (s.e.) | p-value  (R^2^) | Regress Coeff  (s.e.) | p-value  (R^2^) |
| Male^b^ | 0.272  (0.00628) | < 2.2E-308  (0.0345) | 0.304  (0.00909) | 4.24E-239  (0.03293) | 0.250  (0.00919) | 3.59E-159  (0.02645) |
| Age | 0.017  (0.00019) | < 2.2E-308  (0.0809) | 0.017  (0.00038) | < 2.2E-308  (003722) | 0.012  (0.00038) | 4.53E-212  (0.02205) |
| BMI | 0.038  (0.00049) | < 2.2E-308  (0.0708) | 0.052  (0.00080) | < 2.2E-308  (0.08814) | 0.050  (0.00076) | < 2.2E-308  (0.09529) |
| African American^a^ | -0.175  (0.01853) | 2.15E-20  (0.0015) | 0.019  (0.02613) | 3.06E-01  (0.00002) | -0.060  (0.02707) | 3.43E-02  (0.00016) |
| East Asian^a^ | 0.198  (0.01105) | 2.02E-70  (0.0062) | 0.373  (0.01813) | 2.53E-92  (0.01341) | 0.366  (0.01833) | 4.89E-87  (0.01503) |
| Latino/Hispanic^a^ | 0.063  (0.01060) | 7.22E-09  (0.0006) | 0.208  (0.01699) | 1.06E-33  (0.00464) | 0.155  (0.01774) | 1.14E-17  (0.00269) |
| South Asian^a^ | 0.132  (0.04189) | 2.89E-03  (0.0002) | 0.447  (0.06924) | 3.56E-10  (0.00132) | 0.397  (0.06920) | 2.92E-08  (0.00126) |

^a^White/European was set as the reference group

^b^Female was set as the reference group

Table S4: Association of covariates with normalized FG based on all statin users after start of statin, by drug filtering

|  | Filtering | | | | | | | |
| --- | --- | --- | --- | --- | --- | --- | --- | --- |
|  | None | | Diabetic Drugs | | Other Drugs | | All Drugs | |
| Covariates | Regress Coeff  (s.e.) | p-value | Regress  Coeff  (s.e.) | p-value | Regress  Coeff  (s.e.) | p-value | Regress  Coeff  (s.e.) | p-value |
| Male^b^ | 0.158  (.00766) | 3.20E-93 | 0.137  (.00731) | 2.07E-77 | 0.160  (.00816) | 4.49E-84 | 0.144  (.00778) | 3.51E-75 |
| Age | 0.009  (.00033) | 2.50E-181 | 0.011  (.00032) | 1.31E-236 | 0.009  (.00036) | 2.34E-140 | 0.010  (.00035) | 5.25E-169 |
| BMI | 0.038  (.00062) | < 2.2E-308 | 0.038  (.00062) | < 2.2E-308 | 0.038  (.00069) | < 2.2E-308 | 0.037  (.00069) | < 2.2E-308 |
| African American^a^ | -0.101  (.02182) | 8.30E-06 | -0.092  (.02128) | 3.54E-05 | -0.096  (.02369) | 1.03E-04 | -0.097  (.02303) | 5.65E-05 |
| East Asian^a^ | 0.274  (.01508) | 1.30E-72 | 0.265  (.01448) | 1.52E-73 | 0.277  (.01596) | 6.22E-66 | 0.262  (.01534) | 5.76E-64 |
| Latino/  Hispanic^a^ | 0.074  (.01446) | 8.64E-07 | 0.066  (.01405) | 7.51E-06 | 0.075  (.01536) | 2.43E-06 | 0.063  (.01491) | 4.86E-05 |
| South Asian^a^ | 0.221  (.05861) | 3.25E-04 | 0.120  (.05744) | 4.46E-02 | 0.188  (.06118) | 3.60E-03 | 0.107  (.05929) | 7.77E-02 |
| avgFGpreStatin | 0.015  (.00014) | < 2.2E-308 | 0.022  (.00019) | < 2.2E-308 | 0.015  (.00015) | < 2.2E-308 | 0.021  (.00020) | < 2.2E-308 |

^a^White was set as the reference group

^b^Female was set as the reference group

^c^avgFGpreStatin: average FG during the 5 years prior to start of statin

No filtering (no. of subjects = 29747, no. of observations = 157381); Filtered for diabetic drugs (no. of subjects = 28183, no. of observations = 137629); Filtered for other drugs (no. of subjects = 26625, no. of observations = 121015); Filtered for all drugs (no. of subjects = 25153, no. of observations = 106913)

**Supplementary Figures**

Figure S1. Median FG by age group for three subgroups of FG measures

Figure S2. Normalized FG by age group for three subgroups of FG measures, adjusted for BMI, sex and race/ethnicity

Figure S3. Cumulative distribution of total statin exposure (in years) as analyzed among the GERA cohort statin users

Figure S4. FG by duration of statin exposure for statin types lovastatin, simvastatin and atorvastatin (filtering for all drugs); adjusted for age, sex, BMI, race/ethnicity and average FG during the 5 years prior to start of statin

1. Outpatient measures after age 21 [↑](#footnote-ref-1)
2. Cholestyramine/Sucrose, Cholestyramine/Aspartame, Niacin, Colestipol HCL, Colesevelam HCL, Omega-3 Acid Ethyl Esters, Om-3/DHA/EPA/Fish Oil, Probucol, Omega-3-Acid Ethyl Esters, Rare Rx [↑](#footnote-ref-2)
